# Causes and Follow-Up of Central Diabetes Insipidus in Children

**DOI:** 10.1155/2019/5303765

**Published:** 2019-03-27

**Authors:** Wendong Liu, Jing Hou, Xiuqin Liu, Limin Wang, Guimei Li

**Affiliations:** ^1^Department of Pediatrics, Qingdao Municipal Hospital Affiliated to Qingdao University, Qingdao, China; ^2^Department of Pediatrics, Shandong Provincial Hospital Affiliated to Shandong University, Jinan, China

## Abstract

**Objective:**

To identify the causes of central diabetes insipidus (CDI) by evaluating the values of magnetic resonance imaging (MRI) in the diagnosis of pediatric CDI, providing evidence for the clinical diagnosis and treatment of CDI.

**Methods:**

Seventy-nine patients with CDI (CDI group) hospitalized from July 2012 to March 2017 and 43 healthy children (control group) were enrolled in this study. All cases underwent MRI examination including T1-weighted three-dimensional magnetization-prepared rapid gradient-echo (T1WI-3D-MP RAGE) imaging sequences. The pituitary volume, the signal intensity of posterior pituitary, and the morphology of pituitary stalk were measured between two groups. The medical history, urine testing, imaging of hypothalamic-pituitary region, and hormone levels were also recorded.

**Results:**

Age and gender were matched between the CDI and control groups. The height and BMI in the CDI group were less and the urine volume in 24 h was higher than those in the control group. The signal intensity of the posterior pituitary was higher in the control group, whereas the pituitary volume was smaller in the CDI group. In the CDI group, 44 cases presented with morphological changes of the pituitary stalk. Clinical symptoms mainly included polydipsia, polyuria, short stature, and vomiting. All patients were confirmed by water deprivation vasopressin test. Forty-four CDI children were associated with hypopituitarism, including 33 cases of PSIS with multiple pituitary hormone deficiencies (MPHD) and 11 cases of growth hormone deficiency (IGHD). The pituitary volume in the cases of pituitary stalk interruption syndrome (PSIS) with MPHD was smaller than that in the IGHD patients.

**Conclusions:**

The signal intensity ratio of the posterior lobe, pituitary volume, and the morphology of pituitary stalk on T1WI-3D-MP RAGE image contribute to the diagnosis of CDI.

## 1. Introduction

Central diabetes insipidus (CDI) is characterized by polyuria and polydipsia and caused by deficiency of arginine vasopressin (AVP), an antidiuretic hormone which acts on V2 receptors in the kidney to promote reabsorption of free water [1, 2]. Patients with CDI may have anterior pituitary hypofunction, complicated with growth hormone deficiency alone or multiple pituitary hormone deficiencies, leading to abnormalities in the anterior pituitary hormone axis and affecting the pediatric growth and development. Therefore, unravelling the etiology and comprehensive assessment of the condition should be performed for early intervention treatment.

With the development of laboratory examinations and imaging technologies, the diagnosis and etiological identification of CDI have become mature. Magnetic resonance imaging (MRI) of the hypothalamic-pituitary region can visually show the lesions, adjacent structures, and the abnormality of hypothalamic-neuronal system, which plays an irreplaceable role in the diagnosis of CDI [3, 4]. However, due to the small structures of the hypothalamus and pituitary gland, the abnormalities of idiopathic CDI patients cannot be identified with conventional MRI due to low resolution [5]. Nevertheless, T1-weighted three-dimensional magnetization-prepared rapid acquisition gradient-echo (T1WI-3D-MP RAGE) sequence can contribute to the etiological diagnosis of CDI. The T1WI-3D-MP RAGE scan is a newly developed rapid T1-weighted imaging sequence which can be used for continuous thin-slice scanning, reducing partial volume effects and particularly reducing postcranial concave artifact interference [6, 7]. It is helpful to show small lesions in details [8], especially the pituitary stalk and the posterior pituitary. It is simple, feasible, and practical to utilize this sequence to display the hypothalamic-pituitary region. T1WI-3D-MP RAGE sequence is a new sequence, and its clinical application value remains to be confirmed by more studies. The application of this sequence in the hypothalamic-pituitary zone will allow its advantages to be exploited and may provide the basis for the optimization of CDI imaging protocols.

In this study, the saddle area of 79 CDI patients (CDI group) and 43 normal children was collected (control group). The posterior pituitary signal intensity and pituitary stalk diameter were measured and compared between the two groups. The high-resolution T1WI-3D-MP RAGE imaging combined with MRI, analysis of hormonal changes, and long-term follow-up were conducted in children with different causes of CDI, providing an objective basis for timely diagnosis of CDI, assessment of disease condition, and assessment of clinical prognosis.

## 2. Materials and Methods

### 2.1. Subjects

Seventy-nine patients eligible for the criteria of CDI admitted to the Department of Pediatrics and Pediatric Endocrinology, Shandong Provincial Hospital, from July 2012 to March 2017 were selected for the CDI group. Forty-three gender- and age-matched normal children were assigned into the control group.

Inclusion criteria are as follows: (1) polydipsia and polyuria, urine output > 3000 ml/m^2^ within 24 h, or >200 ml/h or >6 ml/kg/h lasting for >24 h; urine specific gravity ≤ 1.005, urine osmotic pressure ≤ 200 mOsm/kg·H_2_O; plasma osmolality ≥ 300 mOsm/kg·H_2_O; urine osmolality/blood osmotic pressure < 1; (2) water deprivation test: dehydration symptoms after 4-6 h of water deprivation, constant urine volume, urine specific gravity ≤ 1.015, urine osmotic pressure ≤ plasma osmotic pressure; (3) pituitary vasopressin test: rapid increase in urine specific gravity ≥ 1.018, urinary osmotic pressure rise > 9%, urine osmotic pressure/plasma osmotic pressure > 1; and (4) plasma vasopressin determination: AVP value less than normal (normal 1-1.5 ng/l).

Exclusion criteria are as follows: (1) trauma- or surgery-induced transient diabetes insipidus; (2) water pressure test prompted for mental polydipsia, NDI; (3) abnormal heart and kidney function; and (4) MRI images that did not meet diagnostic requirements.

### 2.2. MRI Examination

A Siemens 3.0 T superconducting MRI scanner was used. All patients underwent pituitary T1WI-3D-MP RAGE scans with sagittal and coronal T2WI plain scans, and gadolinium diethylenetriamine-pentaacetic acid (Gd-DTPA) as contrast agent was used at a dose of 0.1 mmol/kg. The enhanced scan was performed immediately after pushing the contrast agent. Scan parameters in each sequence for the enhanced MRI examination of the hypothalamic-pituitary region are shown in Table 1.

### 2.3. Data Acquisition and Analysis

The family history, chief complaints, gender, age and duration of onset, water deprivation vasopressin test results, and laboratory test results were recorded for all patients with CDI. The obtained data were analyzed and compared between two groups.

### 2.4. Urine and Blood Tests

24-hour urine volume was monitored, and urine specific gravity, urine osmolality, blood Na^+^, and blood osmotic pressure were measured.

### 2.5. Water Deprivation Test

The water deprivation test can roughly distinguish between CDI and mental polydipsia. Active water restriction was performed for a period of time before the experiment. Complete water deprivation was performed in the evening of the day after the start of the trial, and some subjects could not tolerate water withdrawal from 4 am. The clinical observation of the water deprivation test was performed at 8 o'clock in the morning. The measurement indicators included body weight, heart rate, blood pressure, urine volume, urine specific gravity, urine osmotic pressure, blood Na^+^, and blood osmotic pressure. After water deprivation, the urine output was not significantly reduced, with clear urine, mental irritability, elevated body temperature, weight loss, urine osmotic pressure < osmotic pressure, and elevated blood osmolality (>305 mOsm/kg·H_2_O could terminate the test), who were DI, and urine osmotic pressure of complete diabetes insipidus was less than blood osmotic pressure, and urine osmotic pressure of partial diabetes insipidus was less than blood osmotic pressure. Some children had obvious polyuria and polydipsia during the day, but enuresis nocturna was normal, there were no signs of water shortage, the basic blood sodium was reduced or the normal lower limit, the urine output was reduced after the water deprivation test, the urine became yellow, the urine osmotic pressure and the urine specific gravity were elevated, SG was above 1.018, weight loss was slight, serum sodium and osmotic pressure remained normal, and preliminary consideration was given to psychogenic polydipsia (PP).

### 2.6. Vasopressin Test

Water deprivation vasopressin test combined with MRI was used to identify CDI and NDI. After the water deprivation test, when patient's urine volume, urine specific gravity, and urine osmotic pressure were stable, the patient was injected with 5 units of pituitary vasopressin (children 6 units/m^2^); and the urine volume, urine specific gravity, and urine osmotic pressure were observed. After the injection of vasopressin, urine output in CDI was decreased, urine specific gravity and osmotic pressure were increased, and the response in NDI was not obvious.

### 2.7. Anterior Pituitary Functional Examination and Other Examinations

Anterior pituitary function: (1) two growth hormone (GH) stimulation experiments were done: insulin hypoglycemic GH stimulation test and levodopa (L-DOPA) or arginine (ARG) GH stimulation test. The arginine test (0.5 g/kg, maximum 30 g) could be combined with levodopa (10 mg/kg, maximum 0.5 g), and insulin-induced hypoglycemia (0.075-0.1 u/kg) stimulation test was required for the cortisol challenge test. Blood was drawn at 0, 30, 60, 90, and 120 min, and GH was measured. At 0 min, insulin-like growth factor 1 (IGF-1) was added. After stimulation, GH secretion > 10 *μ*g/l (10 ng/ml) was a normal response, <5 *μ*g/l (5 ng/ml) was complete GH deficiency, and 5-10 ng/ml was partial GH deficiency. (2) Pituitary-thyroid axis function: determination of thyroid-stimulating hormone (TSH), T3, and T4. Patients with free T4 < 12.0 pmol/l and no increase of TSH (TSH < 5 uIU/ml) could be diagnosed as secondary hypothyroidism (TSHD). (3) Pituitary-adrenal axis function: the diagnostic criteria for secondary adrenocortical insufficiency (ACTHD) were that cortisol was less than 138 nmol/l at 8 o'clock in the morning or cortisol was lower than 550 nmol/l in insulin-induced hypoglycemia but adrenocorticotropic hormone (ACTH) was not significantly increased. (4) Pituitary-gonadal axis function: determination of follicle-stimulating hormone (FSH), luteinizing hormone (LH), estradiol (E2), testosterone (TO), and prolactin (PRL). The diagnostic criteria for hypogonadism were that the basal level of gonadotropin (FSH, LH) was lower than the normal detection limit (0.1 mIU/ml) or that the LH peak in the GnRH stimulation test was less than 2.8 mIU/ml, with or without FSH peak < 3.7 mIU/ml; LH peaks in peak adolescents undergoing GnRH stimulation were less than 5 mIU/ml; peak of LH/FSH was less than 0.6. (5) Serum prolactin (PRL) levels: diagnostic criterion for hyperprolactinemia was that PRL was higher than 25 ng/ml; the diagnostic criterion for hypoprolactinemia was that PRL was less than 5 ng/ml.

Patients with space-occupying lesions in the sellar area or thickening of the pituitary stalk should also undergo a systemic radionuclide bone scan to assist in the diagnosis of Langerhans cell histiocytosis (LCH) and be diagnosed with Langerhans cell tissue cells detected by a puncture smear from the skin with a hemorrhagic papule or skeletal lesion. Routine determination of alpha-fetoprotein (AFP), human chorionic gonadotropin (*β*-HCG), and carcinoembryonic antigen (CEA) was used to assist in the diagnosis of germ cell tumors.

### 2.8. Pituitary-Related Index Measurements and Diagnostic Standards on MRI Images

The T1WI-3D-MP RAGE was measured and averaged by three observers, and the image was magnified several times. The measured data were statistically compared between the CDI and control groups.

*(1) Measurement of Pituitary Volume*. The pituitary morphology was observed. In the median sagittal position, the height of the central pituitary and the longest diameter before and after the pituitary were measured. The width of the pituitary was measured at the central coronal position. The volume of the pituitary was calculated based on the volume of the pituitary = 1/2 (long diameter × highdiameter × wide diameter).

*(2) Measurement of Posterior Pituitary Signal*. The position and size of the posterior pituitary were carefully observed, and the posterior pituitary signal intensity and the pons signal were measured in the median sagittal position. The region of interest was located posterior to the pituitary or the inner side of the pons. The ratio of posterior pituitary signal intensity = average gray value of the posterior pituitary/average gray value of pons. The ratio of posterior pituitary signal intensity > 1.00 was defined as a high signal, and ≤1.00 was a high signal loss or decrease. Children with CDI showed high signal loss in the posterior pituitary or a significant reduction in high signal volume.

*(3) Evaluation of Pituitary Stalk Morphology*. The morphology of the pituitary stalk, saddle area, and parasellar structures was observed. Pituitary stalks could be classified to be blocked, normal, and thickened depending on the morphology.

Pituitary stalk block, including pituitary stalk absent and pituitary stalk sessile, was generally assessed by thin-section MRI and clinically known as pituitary stalk block syndrome. Our MRI classification of blockade [9] was mainly based on whether it was divided into two consecutive stages: the pituitary stalk was partially blocked and could be displayed on MRI images, but it was represented by a slender pituitary stalk with or without pituitary ectopic position or missing, mostly accompanied by anterior pituitary dysplasia (the anterior pituitary volume became smaller, and the height was less than 1 standard deviation of the same age group or more); this condition was called partial blockade; the pituitary stalk was completely blocked. The pituitary stalks on the MRI image cannot be displayed, or display was interrupted. The ectopic or absent posterior pituitary and the anterior pituitary hypoplasia (same as before) were called complete blockade.

Tien et al. [10] suggested that the upper limit of the pituitary stalk was 3.5 mm on the pituitary MRI, and the pituitary stalk was thickened if it exceeded this limit. Elster et al. [11] reported that the pituitary stalk may be enlarged when pregnant but no more than 4 mm. We combined the literature to divide the thickening of the pituitary stalk into mild, moderate, and severe degrees according to the degree of thickness [12], of which the slightest thickening was the maximum transverse diameter of the pituitary stalk 3.0-4.5 mm, and the medium thickening was that the maximum transverse diameter in the pituitary stalk was 4.6-6.5 mm, and severe thickening was that the maximum transverse diameter in the pituitary stalk was 6.5 mm or more.

### 2.9. Statistical Analysis

All data were analyzed by using SPSS 17.0 statistical software (SPSS Inc., Chicago, IL, U.S.). The results were expressed as the mean ± standard deviation (SD). The count data were statistically compared using the *χ*^2^ test. The measurement data were statistically compared using the independent sample design *t*-test. A *P* value of less than 0.05 was considered as statistically significant.

## 3. Results

### 3.1. Clinical Features of CDI Patients

Seventy-nine CDI patients eligible for the criteria were included in the study, 53 male and 26 female. The onset age was ranged from 12 months to 15 years, and the disease duration was ranged from 7 months to 9 years. All patients had no family history of CDI, and 25 patients had a history of central nervous system surgery for more than 3 months. The most common symptoms were polydipsia and polyuria in all patients. Seven patients experienced progressive vision loss, loss of vision, paroxysmal headache, and other nonspecific symptoms with mild polydipsia and polyuria. Other symptoms appeared, such as increased nocturia and bedwetting (75 cases), breast and vulvar development (11 cases), short stature (44 cases), anorexia (63 cases), vomiting (25 cases), headache (3 cases), rash (2 cases), emaciation (10 cases), mental retardation (2 cases), and abnormal walking (1 case). All patients were confirmed as having CDI by the water deprivation vasopressin test and MRI of the pituitary area.

### 3.2. Etiological Classification of CDI

**Figure 1:**
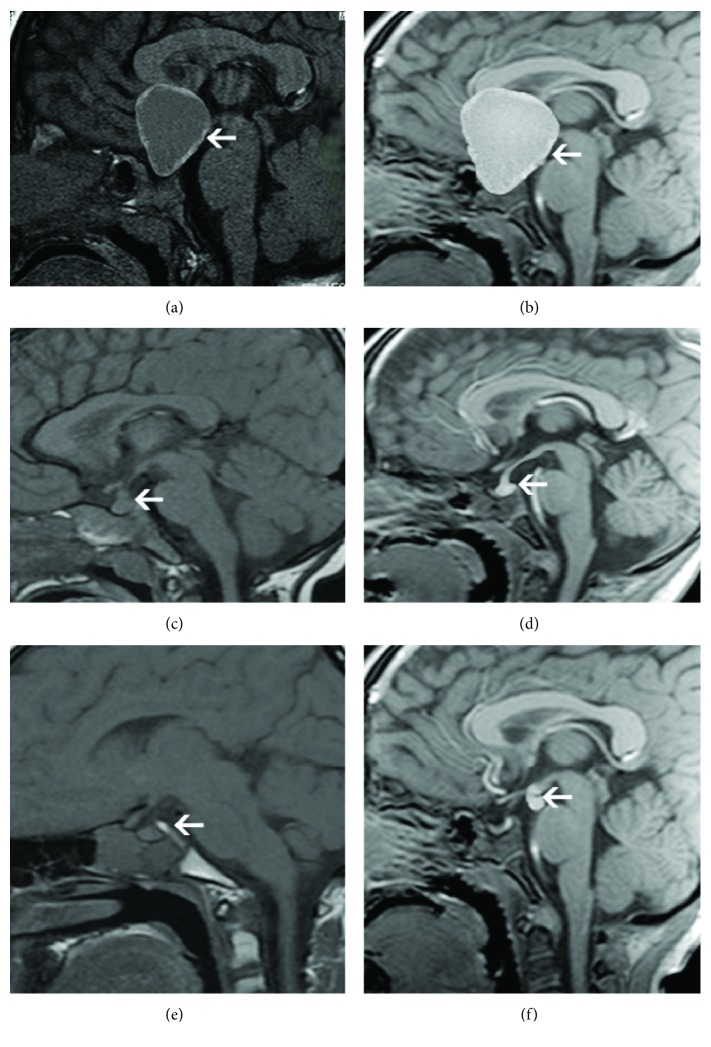
Etiological classification of CDI. Craniopharyngioma: (a) T1WI image showed tumor (white arrowhead); (b) 3D examination showed clearer uniform tumor (white arrow). Intracranial germinoma: (c) T1WI image showed that the upper edge of the pituitary was uplifted, and the posterior high signal was not shown, and the pituitary stalk was thickened (white arrow); (d) the enhanced examination showed that the pituitary stalk and pineal gland were uniformly nodular (white arrows). Langerhans cell histiocytosis: (e) T1WI showed a thickening of the pituitary stalk, showing T1 and other T2 signals, clear edges, pressure in the third ventricle, disappearance of the funnel crypt, slightly increased pressure in the optic chiasm, and no signal in the posterior pituitary; (f) the enhanced scan showed mild even enhancement of the mass, and the anterior pituitary was less clear.

Of the 79 children with CDI, 25 (15 male and 10 female, aged 4-13 years with a median age of 8 years) had craniopharyngioma surgery (ameloblastoma type) more than 3 months after operation. Three cases (12%) were diagnosed with CDI before craniopharyngioma surgery (Figures 1(a) and 1(b)), 20 cases (80%) after craniopharyngioma surgery, accompanied by 3-5 anterior pituitary functions. Before and after radiotherapy for germ cell tumors, 17 cases with intracranial germ cell tumors (icGCT) were followed up, and the pituitary MRI showed that 7 cases were located in the saddle region and the pineal region (Figures 1(c) and 1(d)), and 3 cases were located in the saddle region. The thickening of pituitary stalks disappeared in 7 cases and was rapidly increased during follow-up; tumor markers and postradiotherapy response confirmed icGCT. The levels of *β*-HCG were increased in 4 patients with polydipsia, polyuria, and dwarfism, and the levels of *β*-HCG increased in 4 icGCT patients with polydipsia, polyuria, weight loss, and vomiting. The levels of IGF-1 in 5 cases (29.4%) were decreased, 6 cases (35.3%) showed hypopituitary-thyroid dysfunction, 4 cases (23.5%) appeared to have low secondary cortisol, 2 cases (11.8%) experienced an increase in stress cortisol, 2 cases were treated with surgery, 14 (82.4%) icGCT were sensitive to radiotherapy, and tumors disappeared or nearly disappeared. Eleven cases (64.7%) had 1-5 types of hypopituitarism. Two patients have Langerhans cell histiocytosis (Figures 1(e) and 1(f)), and 33 cases have pituitary stalk interruption syndrome (PSIS). PSIS is a congenital abnormality of the pituitary gland responsible for pituitary deficiency, characterized by the triad of a thin or interrupted pituitary stalk, an ectopic posterior pituitary, and hypoplasia or aplasia of the anterior pituitary on MRI. Pituitary MRI showed that anterior pituitary atrophy, pituitary stalk disruption, and high signal of posterior pituitary disappeared in 30 cases. Ectopic posterior pituitary nearly disappeared in 3 cases, and idiopathic CDI in 2 cases. The pituitary stalk was slightly thickened, and the posterior pituitary gland disappeared. The coagulation treatment was performed and followed up once every 3 months. The pituitary MRI was initially reviewed once every 3 months and reviewed once in the second half of the year. The pituitary stalk returned to normal after 2-year follow-up. Therefore, it was considered as idiopathic CDI.

### 3.3. Water Deprivation Vasopressin Test

The 24 h urine volume of 79 patients was 202.7 ± 9.83 ml/kg. Specific data of water deprivation vasopressin test are shown in Table 2. The urine loss of all patients was not significantly reduced after water deprivation, urine specific gravity and urine osmotic pressure were not significantly increased (all *P* > 0.05), and serum sodium and osmotic pressure were significantly increased (both *P* < 0.05). After subcutaneous injection of vasopressin aqueous solution in posterior pituitary, urine volume was evidently decreased, urine specific gravity and urine osmotic pressure were considerably increased, and serum sodium and osmotic pressure were dramatically decreased (all *P* < 0.05).

### 3.4. Comparison of MRI Findings between CDI and Control Groups

No significant difference was noted in age and gender between the two groups. However, height and body mass index (BMI) in the CDI group were lower than those in the control group. The 24 h urine volume was higher in the CDI group than in the control group (Table 3). Of these, 44/79 cases (55.6%) were eligible for the criteria for dwarfism.

**Figure 2:**
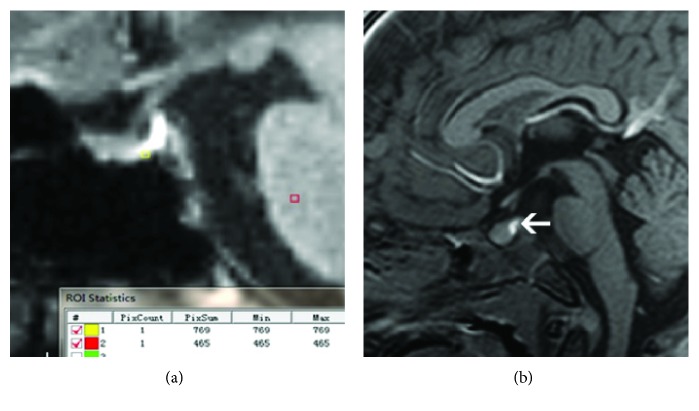
Measurement of the posterior pituitary signal intensity. (a) The signal intensity of the posterior pituitary of the normal pituitary was higher than that of the pons. (b) The high signal of the posterior pituitary (white arrow) contrasts with the anterior pituitary and the pons.

The ratio of the posterior pituitary signal intensity was 1.68 ± 0.25 in the control group and 0.88 ± 0.21 in the CDI group. The high signals of the posterior pituitary in CDI patients almost disappeared or were decreased (Figure 2), and most of the signals were low signals relative to the pons. The pituitary volume was roughly represented by the pituitary height, width, and front-rear diameter (mm^3^). The pituitary volume in 43 patients was 320.8 ± 64.5 mm^3^ in the control group; 35 patients in the CDI group have 160.2 ± 30.4 mm^3^. The pituitary volume of 33/35 cases (94.3%) of patients with CDI had different degrees that became smaller (*P* < 0.01).

**Figure 3:**
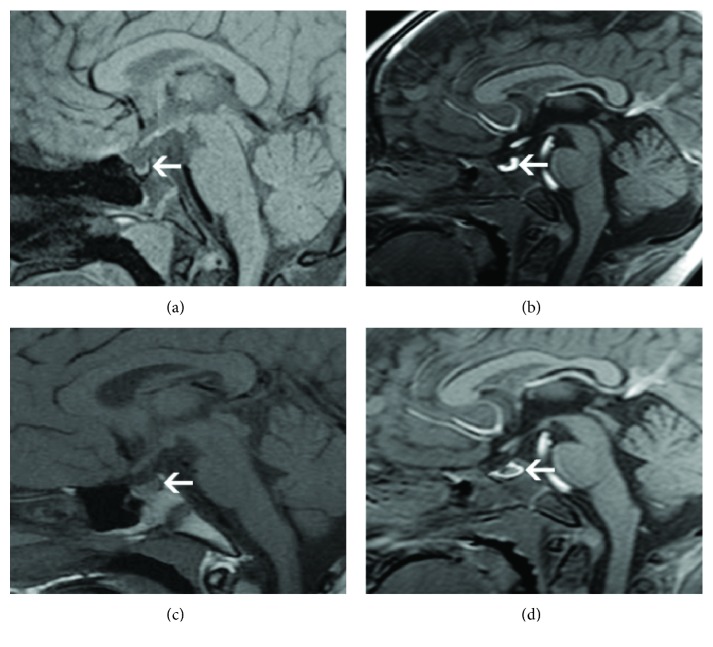
Pituitary stem blockage syndrome. (a) T1WI showed that the anterior pituitary was thin, the central site was sunken, the posterior pituitary high signal was not clearly shown, and the pituitary stalk was interrupted (white arrow). (b) The enhanced examination showed a small ectopic position in the anterior pituitary and after the optic chiasm, and obvious uniform enhancement was found in the posterior leaves. (c) T1WI showed that high intensity of posterior pituitary was disappeared and the pituitary stalk showed a thin line. (d) The enhanced examination showed anterior pituitary atrophy.

In 79 patients, except for 25 cases after craniopharyngioma surgery, pituitary iccCT in the saddle area could not be identified in 11 cases, and pituitary stalk abnormalities were found in 44 cases, among which 33 cases of PSIS patients had complete occlusion of the pituitary stalk, and 3 cases were partially blocked (Figure 3). Pituitary stalks in 11 cases (two cases were slightly thickened and were confirmed as idiopathic CDI after long-term follow-up, 5 cases were moderately thickened, and 4 cases were severely thickened) were thickened to varying degrees. As for 9 cases with severe thickening of the pituitary stalk, 7 cases showed rapid growth and multiple pituitary hormone deficiencies (MPHD), whose tumor markers were positive and tumors disappeared after radiotherapy and confirmed as germ cell tumor. Two cases were diagnosed with skull defects, confirmed as Langerhans cell histiocytosis by pathological examination. The pituitary stalk thickening was reduced after chemotherapy, but polydipsia and polyuria should be properly controlled.

### 3.5. MRI Changes of CDI Complicated with Hypopituitarism

The abnormalities of hormone secretion in the anterior pituitary were found in 44 cases upon the initial diagnosis (Table 4). The anterior leaf volume of 33 patients with MPHD was significantly smaller compared with that of the patients with IGHD (*P* < 0.05). All 44 patients with short stature had growth hormone deficiency, and 2 patients with mental retardation experienced MPHD. As shown in Table 5, 7 cases with moderate-severe pituitary stalk thickening developed into icGCT in the follow-up and appeared as MPHD, and 33 PSIS patients with CDI had MPHD.

## 4. Discussion

Recent studies found that DI was due to hypothalamic-neuronal lesions caused by the lack of different levels of antidiuretic hormone (ADH) or due to kidney sensitivity to AVP deficiency caused by a variety of lesions [13, 14]. In pediatric CDI, etiology diagnosis and pituitary function monitoring are usually delayed [15]. If the diagnosis and treatment of DI is not timely, it will affect the growth and development of children; it will also affect the study and work efficiency of adults, reduce the quality of life, and cause damage to kidney function. Therefore, for CDI, it is necessary to make a clear diagnosis classification, find the cause, and undergo timely symptomatic treatment.

Clinical manifestations of CDI include polydipsia induced by lack of AVP. As for a vast majority of patients, polydipsia is the first symptom, and 79 cases presented with polydipsia in this study. Most patients initially had increased initial frequency of urination and increased urine output, followed by polydipsia. In children with onset of illness, the bladder, ureters, and pelvis may dilate due to prolonged polyuria, impairing kidney function and possibly develop osteoporosis. Children may have nocturia and bedwetting in this study. If the patient is unable to drink water, hypovolemia can occur, such as palpitations, decreased blood pressure, cold extremities, shock, and prerenal azotemia, and timely supplementation of blood volume can be promptly corrected. If the hypovolemic state cannot be corrected promptly, headaches, irritability, hopelessness, and coma can occur. Others included 11 cases with breast and exogenous development. Most children with CDI had digestive symptoms, vomiting and poor appetite. In this study, 63 cases presented with anorexia, 25 cases with vomiting, and 10 cases with weight loss, and CDI patients may be complicated with hypopituitary dysfunction, which may affect the growth and development of children. In this study, 44 cases experienced short stature, and 2 cases suffered from mental retardation.

GH and GnRH deficiencies often occur before the anterior pituitary hormone deficiency, subsequently followed by TSH and ACTH deficiency [16, 17]. For 79 CDI patients in this study, 44 cases had hypopituitary dysfunction, and all presented with GH deficiency. Among them, 33 cases had two or more hormone deficiencies, 33 cases had secondary hypothyroidism, and 20 cases had hypogonadism. 30 children had secondary adrenal insufficiency, 33 cases had elevated PRL, and 11 cases had reduced PRL, indicating that CDI patients had a variety of endocrine gland secretion abnormalities. Insufficient secretion of hormones in the anterior pituitary gland will produce a series of symptoms and signs of corresponding target gland dysfunction, and its severity is related to the degree of hormone deficiency. This study also found that the pituitary volume of the CDI group was smaller than that of the control group, indicating that when hypothalamic-nerve pituitary system lesions occurred, it not only induced ADH deficiency to cause DI but also induced pituitary anterior pituitary dysfunction. And 44 patients with hypopituitarism in the pituitary gland had organic lesions. Among them, 44 patients with short stature had GH deficiency, and 2 patients with mental retardation also had MPHD. Therefore, patients with CDI needed to pay close attention to the anterior pituitary secretion to actively remove the cause or perform symptomatic treatment and to avoid serious consequences such as short stature and delayed mental development.

When the morphological changes (such as interruption, tumor compression, infiltration, and destruction) of pituitary stalk occur, it can cause the downward transport channel of the pituitary hormone to be blocked, possibly leading to the occurrence of pituitary hypofunction. Biopsy should be performed when the pituitary stalk thickens >6.5 mm to determine the specific cause [18]. Germ cell tumors contain large epithelial cells and small lymphocytes pathologically. They are very sensitive to radiotherapy and chemotherapy and are a curable tumor [19]. When the site of lesion growth is special and difficult to take biopsy, germ cell tumor can be diagnosed based on patient's medical history, serum or cerebrospinal fluid tumor marker detection, and germ cell tumor sensitivity to radiotherapy and chemotherapy [20]. Our study found that 44 CDI patients with anterior pituitary hypothyroidism had pituitary stalk morphological changes, 33 cases showed pituitary stalk block, and 11 cases presented with the thickening of the pituitary stalk. When the pituitary stalk was significantly thickened, the pituitary stalk pathway will be seriously blocked, or when the hypothalamus was under pressure and invasion, the anterior pituitary gland cannot absorb sufficient nutrition and regulation, resulting in low secretion function, atrophy, and lack of a variety of pituitary hormone secretion. The pituitary stalk was severely thickened in 7 cases with sellar region germ cell tumors complicated with MPHD. 2 LCH children also showed severe thickening of the pituitary stalk with MPHD. 2 cases with idiopathic CDI in children showed mild thickening of the pituitary stalk.

Studies have shown that CDI is also an autoimmune disease [21–23]. Study found that autoreactive CD8+ T cells can attack CNS neurons, leading to CDI [24], suggesting that immunotherapy interventions or inhibition of the inflammatory response mediated by the immune system could be used for the treatment of CDI. Previous study has found that a small number of CDI had a family history of antidiuretic hormone due to gene mutation of antidiuretic hormone-posterior leaf hormone transporter (AVP-NPII) [25].

In conclusion, the high signal intensity of the posterior lobe and the pituitary stalk is clearly displayed on T1WI-3D MP RAGE images. The signal intensity ratio of the posterior lobe, the measurement of pituitary volume, and the morphology assessment of pituitary stalk on T1WI-3D MP RAGE images contribute to the diagnosis of CDI. The thickening of the pituitary stalk in patients with CDI should be subject to long-term follow-up. In addition, the detection of tumor biomarkers, such as beta-HCG, plays a role in identifying the etiology of CDI. The premature outcomes in this study remain to be validated by subsequent investigations, such as antibody detection experiment.

## Figures and Tables

**Table 1:** Scan parameters of each sequence for MRI examination.

	TR (ms)	TE (ms)	FA	Thickness/layer spacing (mm)	Matrix	FOV	Scanning time (min)
T1WI-3D-MP RAGE	10.5	4.0	12°	1/0	256 × 256	256 × 256	10.0
T2WI sweep	4000.0	100.0	—	3/0	256 × 256	256 × 256	2.6
T1 enhanced inspection	200.0	9.1	—	3/0	256 × 256	256 × 256	1.2

Abbreviation: TR: time repetition; TE: time echo; FOV: field of view.

**Table 2:** Results of water deprivation and vasopressin test in patients with CID.

	Blood Na^+^ (mmol/l)	Blood osmotic pressure (mOsm/kg)	Urinary osmotic pressure (mOsm/kg)	Urine specific gravity
Before water deprivation	144.3 ± 10.9	286.2 ± 15.6	128.6 ± 6.8	1.00-1.004
After water deprivation	156.0 ± 15.2^∗^	307.5 ± 20.9^∗^	179.1 ± 10.5	1.00-1.010^∗^
After injection of vasopressin	142.9 ± 8.8^∗^	278.7 ± 11.3^∗^	602 ± 58.6^∗^	1.01-1.028^∗^

^∗^ *P* < 0.05.

**Table 3:** Comparison between baseline data of CDI and control groups.

	CDI group (*n* = 79)	Control group (*n* = 43)	*P*
Male/female	53/26	28/15	>0.05
Age	7.60 ± 2.49	7.45 ± 1.96	>0.05
Height (mean ± standard deviation)	-3.23 ± 1.24	0.68 ± 0.35	<0.01
BMI	14.12 ± 2.56	18.28 ± 3.32	<0.05
24 h urine volume (ml/kg)	202.7 ± 9.83	88.6 ± 12.99	<0.01

**Table 4:** Relationship of abnormal hormone secretion in the anterior pituitary with pituitary volume and patient development.

	Number	Pituitary volume		Short stature	Mental retardation
		Smaller	Normal		
IGHD	11	5	6	11	0
MPHD	33	33	0	33	2

**Table 5:** Relationship of pituitary stalk abnormalities with anterior pituitary hormone secretion abnormalities.

Pituitary stalk form	Number	Anterior pituitary hormone		MPHD
		Normal	Deficiency	
Mild thickening	2	2	0	0
Moderate-severe thickening	9	2	7	7
Partially blocked	3	0	3	3
Completely blocked	30	0	30	30

## Data Availability

The data used to support the findings of this study are available from the corresponding author upon request.
